# A Case of Sudden Very Late Onset of Severe Clozapine-induced Agranulocytosis Following an Influenza Vaccine

**DOI:** 10.7759/cureus.4682

**Published:** 2019-05-16

**Authors:** Soluny Jean, Louvens Romain, Senders Florvil, Fritz G Heyliger, Amrendra Mandal

**Affiliations:** 1 Internal Medicine, Interfaith Medical Center, Brooklyn, USA; 2 Internal Medicine, State University Hospital of Haiti, Port-au-Prince, HTI

**Keywords:** clozapine-induced agranulocytosis, neutropenia, agranulocytosis and influenza vaccine, clozapine, agranulocytosis, neutropenia and influenza vaccine

## Abstract

Agranulocytosis can be a life-threatening condition because of its high risk of serious infection. It is extremely rare, with an incidence of one to five cases per million in the population per year. About 70% of the cases are associated with medications. Clozapine-induced leukopenia is a well-known clinical entity, justifying regular hematologic surveillance.

Most cases of clozapine-induced neutropenia and agranulocytosis occur during the first three months of treatment. It’s extremely rare after the first year of treatment. However, we present the case of a late-onset of sudden severe agranulocytosis following an influenza vaccine, after more than 156 months of stable neutrophil counts on clozapine.

Clinicians must keep in mind that this complication can occur at any time during the treatment course and may wish to increase the frequency of hematologic surveillance following an influenza vaccine or even consider a risk-benefit approach. Considering the importance of the influenza vaccination and the seriousness of agranulocytosis, further studies are needed to elucidate a potential increase in the risk of severe neutropenia in patients on clozapine receiving the influenza vaccine.

## Introduction

Agranulocytosis, defined as an absolute neutrophil count (ANC) of less than 500 cells/microliter (uL), can be a life-threatening condition [1]. It is associated with an increased risk of significant infection. It is extremely rare, with an incidence of one to five cases per million population per year [2]. Medications are responsible for about 70% of the cases [3]. Clozapine is an atypical antipsychotic used for the treatment of schizophrenia resistant to other antipsychotic drugs. It is a well-known cause of neutropenia, which is why all patients on this drug have strict regular hematologic surveillance.

Most cases of clozapine-induced neutropenia and agranulocytosis occur during the first three months of treatment, with some cases between three months and one year. It’s extremely rare after the first year of treatment [4-5]. However, we present the case of a very late-onset of sudden severe agranulocytosis following the influenza vaccine, after more than 156 months of stable neutrophil counts on clozapine.

## Case presentation

**Figure 1:**
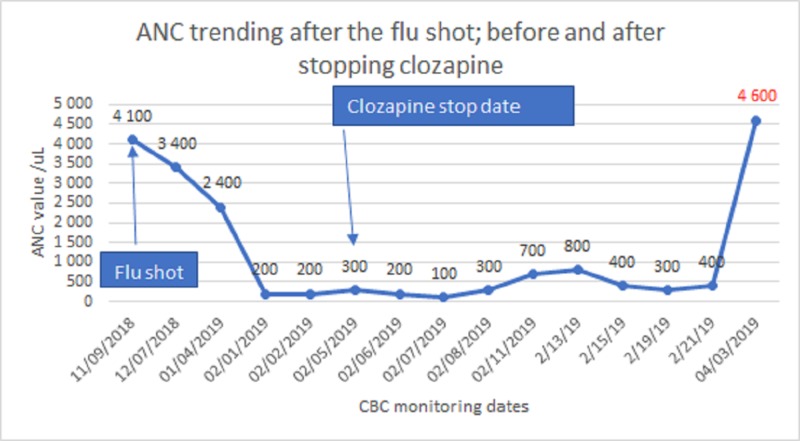
Timing of the flu shot and ANC trending before and after stopping clozapine ANC: absolute neutrophil count; CBC: complete blood count

A 56-year-old African-American human immunodeficiency virus (HIV)-negative male patient presented to the general medicine clinic with an acute drop of his white blood count (WBC). His past medical history (PMH) included hypertension, type 2 diabetes mellitus, chronic obstructive pulmonary disease (COPD), and a history of schizophrenia for which he had been treated with clozapine (Clozaril) for more than 13 years. On his monthly complete blood count (CBC), the ANC was 3,400 cells/uL (normal value: 2,000 - 7,900 cells/uL) and 2,400 cells/uL one and two months prior to his presentation, respectively. Suddenly, at the current presentation, his ANC was reported to be 200 cells/uL (Figure 1).

His active medications were simvastatin, metoprolol, lisinopril, aspirin, metformin, haloperidol, and benztropine, all of which he had been taking for more than five years with no adverse effect. On further questioning, the only recent new event was his influenza vaccine about seven weeks prior.

Review of systems was negative for any new symptoms, including fever, chills, asthenia, cough, or sore throat. Physical examination was unremarkable. His blood pressure was 136/99 mm Hg, the heart rate was 86 beats/min, and his temperature was 97.2° F. He was sent to the emergency department and was admitted. An HIV test, urine toxicology, and a complete metabolic panel were all unremarkable. The clozapine was stopped. The next day, he was cleared by the hematology team for discharge to follow-up as an outpatient.

His ANC was monitored closely after stopping the clozapine and the trend is shown in Figure 1. Seven weeks later, his ANC normalized to 4,600 cells/uL, but he had a relapse of psychosis and had to be admitted to the psychiatric unit.

## Discussion

Agranulocytosis is defined as an ANC < 500 cells/uL. Affected patients are at high risk for life-threatening infections. An increased risk of drug-induced neutropenia (not specifically clozapine-induced neutropenia) has been found to be associated with advanced age (> 50 years old), female sex, and underlying autoimmune disease. Although data are lacking about the risk factors for clozapine-induced leukopenia, few studies have found an increased risk when used concomitantly with other drugs known to cause neutropenia, such as carbamazepine. Clozapine-induced leukopenia (ANC < 1,500) is a relatively common known adverse effect, whereas agranulocytosis is extremely rare [1]. 

When clozapine became available in the United States, weekly hematologic surveillance was the rule. Over the years, it has been proven that the first three to six months are the most dangerous. As per the current recommendations, any patient who is being started on this drug must have a baseline ANC of 1,500 cells/uL (1,000 for a patient with benign ethnic neutropenia). As per the clozapine risk evaluation and mitigation strategy (REMS), weekly monitoring of ANC during the first six months, then every two weeks for the second six months, are required [6]. Thereafter, the CBC will be repeated monthly for the duration of treatment. Our patient had been monitored according to these guidelines. For more than 13 years of treatment, his WBC has been stable.

In several studies, it has been shown that almost all the cases of neutropenia/agranulocytosis occurs within one year after starting treatment, with more than 80% during the first three to six months [4-5]. After more than 13 years of a stable white count, our patient presented with sudden severe agranulocytosis. Within two months, his ANC dropped from 3,400 to 100 cells/uL with intact platelets and red blood cells.

After an exhaustive history and review of the patient chart, the only new event preceding the decline in WBC was the influenza vaccine in the previous seven weeks. Seven weeks after stopping clozapine, the ANC returned to normal. After the flu vaccine, there was a constant drop in the ANC. The likelihood that the flu vaccine was the triggering factor was considered because the patient had been stable for more than a decade on clozapine and suddenly experienced this drastic decline in ANC after being vaccinated. There is one case report in the literature of severe neutropenia three weeks after an influenza vaccine. This case was followed by a prospective study of 70 people (not on clozapine) after they received the flu shot. There were no significant changes in the levels of hemoglobin, neutrophils, monocytes, eosinophils, or platelets after vaccination, but the total WBC counts were significantly lower at four weeks than at baseline [7]. The decrease in the WBC count revealed in this study could probably become more significant for a patient with important risk factors for neutropenia, such as taking clozapine.

In view of the event sequence, it is reasonable to think that the influenza vaccine might have been the triggering event for the severe agranulocytosis in our patient taking clozapine. Clinicians might need to consider increasing the frequency of hematological surveillance for their patients on clozapine after receiving the flu vaccine. It might even be justified to consider a risk-benefit approach when it comes to the influenza vaccine for this patient population. Considering the importance of influenza vaccination and the seriousness of agranulocytosis in patients on clozapine, further studies are needed to elucidate an eventual association.

## Conclusions

Clinicians must keep in mind that agranulocytosis can occur at any time during the treatment course with clozapine and may wish to increase the frequency of hematologic surveillance following an influenza vaccine or even consider a risk-benefit approach. Considering the importance of the influenza vaccination and the seriousness of agranulocytosis, further studies are needed to elucidate a potential increase in the risk of severe neutropenia in patients on clozapine who take the influenza vaccine.
